# Identification of Prognostic Biomarker Candidates Associated With Melanoma Using High-Dimensional Genomic Data

**DOI:** 10.3389/fgene.2021.707105

**Published:** 2021-09-13

**Authors:** Brody Kutt, Rachel Burdorf, Travaughn Bain, Nicardo Cameron, Alexia Pearah, Ersoy Subasi, David J. Carroll, Lisa K. Moore, Munevver Mine Subasi

**Affiliations:** ^1^School of Mathematical Sciences, Rochester Institute of Technology, Rochester, NY, United States; ^2^Department of Biomedical and Chemical Engineering and Sciences, Florida Institute of Technology, Melbourne, FL, United States; ^3^Biology Department, Colorado College, Colorado Springs, CO, United States; ^4^Department of Mathematical Sciences, Florida Institute of Technology, Melbourne, FL, United States; ^5^College of Aeronautics, Florida Institute of Technology, Melbourne, FL, United States; ^6^Department of Biochemistry and Molecular Genetics, Midwestern University, Glendale, AZ, United States

**Keywords:** melanoma, gene expression, classification, clustering, feature selection, machine learning

## Abstract

Survival of patients with metastatic melanoma varies widely. Melanoma is a highly proliferative, chemo-resistant disease. With the recent availability of immunotherapies such as checkpoint inhibitors, durable response rates have improved but are often still limited to 2–3 years. Response rates to treatment range from 30 to 45% with combination therapy however no improvement in overall survival is frequently observed. Of the available therapies, many have targeted the BRAFV600E mutation that results in abnormal MAPK pathway activation which is important for regulating cell proliferation. Immune checkpoint inhibitors such as anti-PD-1 and anti-PD-L1 offer better success but response rates are still low. Identifying biomarkers to better target those who will respond and identify the right combination of treatment is the best approach. In this study, we utilize data from the Cancer Cell Line Encyclopedia (CCLE), including 62 samples, to examine features of gene expression (19K+) and copy number (20K+) in the melanoma cell lines. We perform a clustering analysis on the feature set to assess genetically similarity among the cell lines. We then discover which specific genes and combinations thereof maximize cluster density. We design a feature selection approach for high-dimensional datasets that integrates multiple disparate machine learning techniques into one cohesive pipeline. Our approach provides a small subset of genes that can accurately distinguish between the clusters of melanoma cell lines across multiple types of classifiers. In particular, we find only the 15 highest ranked genes among the original 19 K are necessary to achieve perfect or near-perfect test split classification performance. Of these 15 genes, some are known to be linked to melanoma or other cancer progressions, while others have not previously been linked to melanoma and are of interest for further examination.

## 1. Introduction

Melanoma can be a devastating disease, one in which incidence is on the rise, and treatment options are limited for the most aggressive forms of the disease. Several gene mutations are widely expressed in melanoma incidences. In particular, the BRAFV600E mutation occurs in ~60% of patients. This mutation results in constitutive activation of BRAF signaling in the ERK/MAP Kinase pathway with a result of increased cell proliferation and survival (Ascierto et al., 2012). Some of the most promising treatments have been developed as selective inhibitors of this or other pathways. However, not all patients exhibit mutations in these pathways and, of those that do, not all respond to these therapies. If they respond initially, they may also not be responsive upon disease recurrence. Malignant melanoma has shown poor durability overall in response to available treatments. This raises the question of what other factors drive treatment response, tumor aggressiveness, and the ability to metastasize from the original primary lesion site. By identifying subset populations of melanoma as distinguished by gene expression, we can gain a greater understanding of the genomic intravariability in melanoma as well as illuminate potential gene therapy targets enabling a patient-powered precision medicine approach to treatment.

One approach to address this question is to profile gene expression using CDNA microarray screening or transcriptome analyses from patient tumor libraries. These have the advantage of providing known information on tumor response to different therapies, tumor staging, prognosis, and survival data. The downside to this approach is limited access to tissues, tissue heterogeneity, and overall tissue availability (Ryu and et al., 2007). Other approaches include the evaluation of melanoma cell line data. Having increased availability of open-access databases with patient and cell line datasets makes this possible. Some related examples of analyses on this kind of data have been to use hierarchical clustering, similarity core analysis, and Elastic Net regression (Ryu and et al., 2007; Garnett and et al., 2012; Covell, 2015; Rambov and et al., 2015). These methods have had some success in the ability to identify potential genes that may be involved in the upregulation of cell proliferation, drug response, and propensity for metastasis to distant sites. Success has been limited due to the inability to have a good correlation of results from one approach to the next. It is clear that general classes of gene products are identifiable, those being effectors of the cell cycle, its checkpoints, apoptosis, cell adhesion, tumor suppressors, and DNA repair. The availability of data through publicly accessible large databases has provided the ability for tailored approaches to data mining and machine learning. Numerous different approaches are highlighted in the literature often as an effort to assess drug sensitivity data with pathway and gene expression clustering (Garnett and et al., 2012; Brubaker et al., 2014; Jang et al., 2014; Covell, 2015). The Cancer Cell Line Encyclopedia (CCLE) (Barretina et al., 2012; Cancer Cell Line Encyclopedia Consortium and Genomics of Drug, 2015) is one such database in which gene expression, copy number, and drug response data are available for over 1,000 cancer cell lines (http://www.broadinstitute.org/ccle/home). Having large datasets available can provide a powerful tool for identifying pathways and gene expressions that are critical in determining prognosis and overall sensitivity to treatments. In this study, we have selected the melanoma cell lines from the high-dimensional CCLE dataset and used various machine learning methods to identify genes of interest that distinguish these 62 cell lines into three discrete clusters. To date, the use of biomarker data from these databases has provided limited success and will require confirmation in biologic systems to determine true correlative benefits. The overall hope is that these analyses will lead to the development of molecular signatures for targeted therapies and the ability to individualize the treatments for the most durable response.

## 2. Clustering of Melanoma Cell Lines

We used gene expression and copy number features for 62 melanoma cell lines from the CCLE to systematically assess features of interest for distinguishing genomic clusters of melanoma cell lines. CCLE skin cancer data included expression for >19,000 genes, copy number for >20,000 genes and 24 drug sensitivity features. To identify the distinct clusters of the cell lines, we applied the k-means++ (Arthur and Vassilvitskii, 2007) method with feature sets consisting of gene expressions, copy number variations, and their combination. The *k*-means method is a widely-used clustering technique that seeks to minimize the average squared distance between points in the same cluster. Although it offers no accuracy guarantees, its simplicity and speed are very appealing in practice. The *k*-means++ method (Arthur and Vassilvitskii, 2007) introduces an algorithm that is *O*(log*k*)-competitive with the optimal clustering obtained by augmenting *k*-means with an improved initialization technique. Computational experiments showed that the augmentation improves both the speed and the accuracy of *k*-means, often quite dramatically. Likely due to the extreme scarcity and sparseness of our data, more complex clustering techniques did not prove useful.

When plotting a goodness-of-fit metric against the number of clusters specified for the k-means++ algorithm, we can search for a specific number of clusters, *k*, that corresponds to an abrupt shift in the slope of the error curve—called an elbow-point. The goodness-of-fit metric we use is the within-cluster sum of squares (WCSS) which reflects the sum of Euclidean distances of cluster members to cluster centers. An elbow-point with this metric suggests a natural *k* in the data such that the clustering explains a large percentage of variability in the data with a small *k*. Each data point corresponding to each candidate *k* reflects the results of ten randomized trials. The solution with the minimum error is selected.

**Figure 1:**
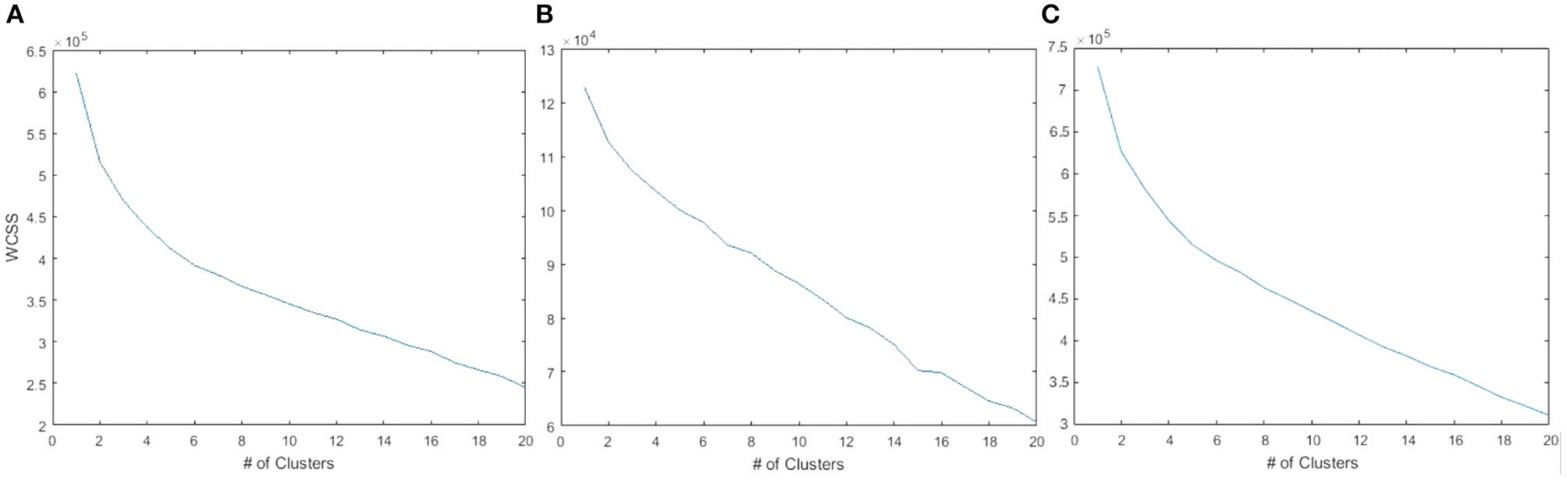
WCSS error plotted against the number of cell clusters. **(A)** 19 K gene expression features showing an elbow-point at approximately five clusters. **(B)** 20 K copy number variation features did not show a clear point of inflection. **(C)** The combined features of gene expression and copy number variation was similar to gene expression alone.

**Figure 2:**
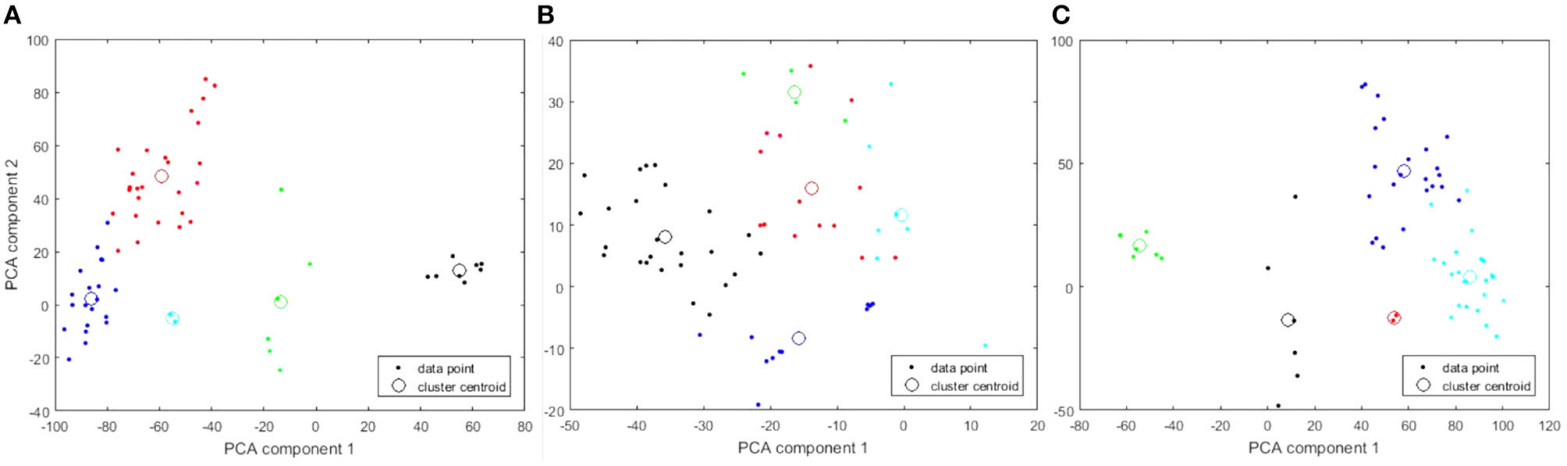
PCA plots of 5-cluster analysis to show how the individual cell lines cluster. Each cluster is identified in a different color. The centroid of each cluster is shown with a large open circle. **(A)** 19 K gene expression features. **(B)** 20 K copy number variation features. **(C)** Combined features.

As shown in Figure 1A, an elbow-point at approximately five clusters exists when considering the gene expression features alone. In Figure 1B, there was no clear suggestion for the optimal *k* when considering the copy number variation features alone indicated by a near-straight line in the graph. Combining the copy number variations with the gene expressions, shown in Figure 1C, did not affect the elbow-point seen in Figure 1A nor change the membership of the resulting cluster indices. This suggests that the copy number variation features are poor candidates for diversifying the cell lines. Principal component analysis (PCA) plots corresponding to all three 5-cluster analyses are presented in Figure 2.

**Figure 3:**
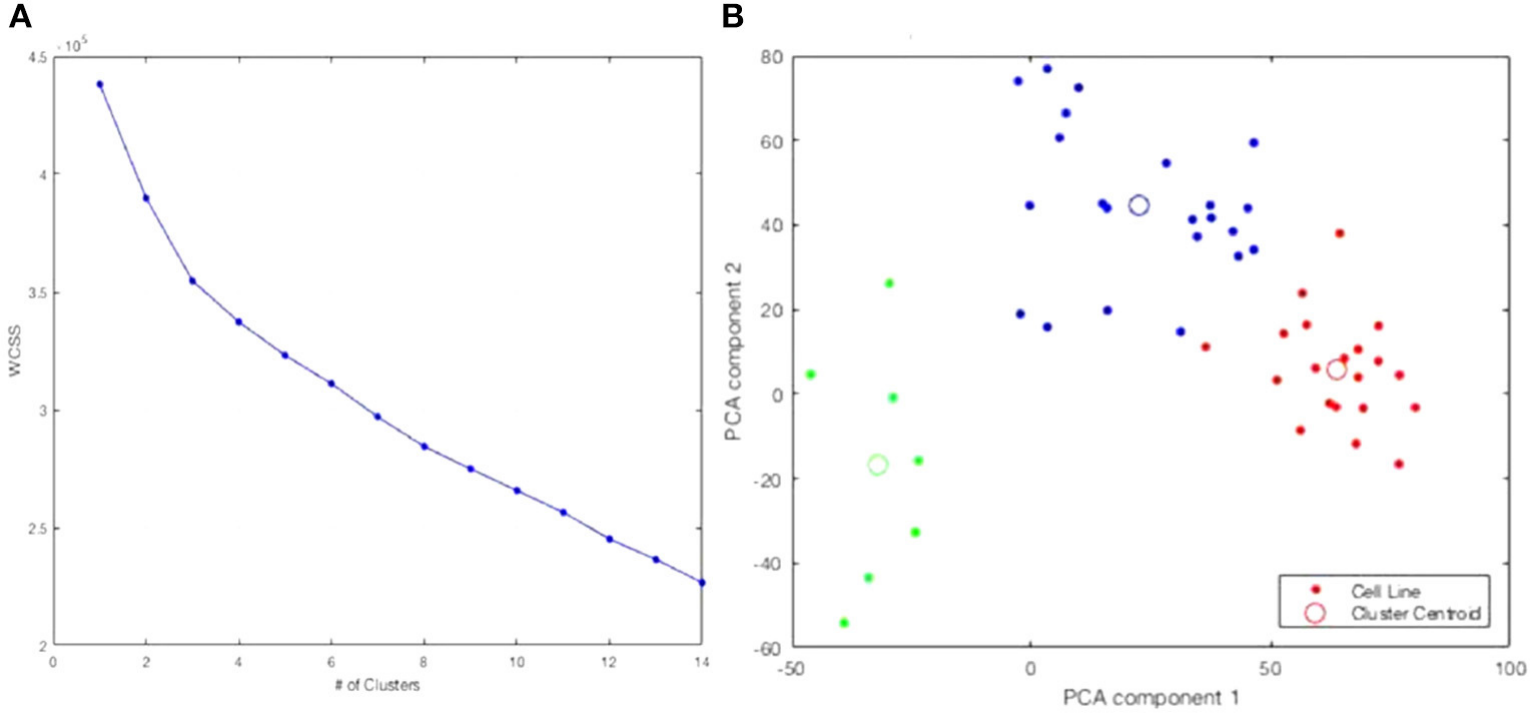
Re-analysis of the 49 cell lines identifies three clusters. **(A)** WCSS error. **(B)** PCA plot of 3-clusters of 49 cell lines with 19 K gene expression features: Cluster 1, Cluster 2, Cluster 3.

Information from the American Type Culture Collection (ATCC) suggested the origin of the two cell lines exclusively clustered together in Figures 2A,C were from a single patient. A different cluster of cell lines originated from The Naval Biosciences Laboratory (NBL) collection. Some of those cell lines were removed from the ATCC and aren't fully characterized for morphology or purity. As a result, we removed these two clusters from further analysis. After removal, 49 cell lines remained. Moreover, considering the results across Figures 1, 2, we continued our investigations on the 49 cell lines with the 19 K gene expression features only. Figure 3 displays the WCSS error and PCA plot of the three clusters of 49 cell lines based on gene expression features.

## 3. Feature Selection and Classification

To identify the patterns of gene expressions best separating the three clusters, we applied a pipeline of various feature selection and classification methods with cluster index used as the response variable. We tailored our methods to work with our high-dimensional dataset.

### 3.1. Identification of Most Significant Gene Expressions

Feature selection methods consist of three main groups: filter, wrapper, and embedded methods. Filter methods rank each feature according to some univariate metric with the response variable. Wrapper algorithms search for the best subset of features concerning the classification performance of an underlying model. The wrapper algorithm typically treats the classification algorithm as a black box, such that any classifier is suitable for the wrapper. One can use standard optimization techniques (hill-climbing, simulated annealing, or genetic algorithms) as the wrapper algorithm. Embedded methods search among different feature subsets, but unlike wrappers, the process is embedded into a classification model and takes place naturally as a part of learning the classifier. Decision Trees are a famous example of a classification algorithm with embedded feature selection properties. For more information on feature selection methods and their applications to genomic and proteomic data, refer to Dubitz (2007) and the references therein.

In part because of the high dimensionality and sparseness of the CCLE melanoma data, one is often led to finding entirely different results from applying feature selection on randomized trials or between different algorithms. The results depend entirely on algorithm details and not the structures within the data itself. Also, in part because of the power of various classification algorithms: using a large enough subset of features, even if they are simply randomly selected, can yield good classification accuracy. The classifier can learn patterns in the data that are circumstantial artifacts as opposed to biologically relevant information. We propose that the most general features which distinguish the data will score well across different kinds of feature selection algorithms and will also yield high classification performances across many different types of classifiers. To reach this end, we first employ a statistical univariate technique, called the Fisher Score (Aggarwal, 2014), and a multivariate SVM-based technique, called Support Vector Machine Recursive Feature Elimination Correlation Bias Reduction (Yan and Zhang, 2015), abbreviated as SVM-RFE+CBR, to reduce the feature set size. These reduce the feature set size to a manageable level for running 500 randomized trials of a search algorithm, called Sequential Floating Forward Search (Pudil et al., 1994), abbreviated as SFFS, wrapped around the *k*-nearest neighbors (KNN) algorithm. Specifically, the objective function of the search is the average test set classification performance of KNN when *k* is varied from three to nine. We randomize the training and test splits for each trial in the last step. Each random trial of the search algorithm returns a 20 feature subset of the 500 feature pool. We record the features which occur in the results the most often. This procedure consolidates the results from three highly varied algorithms.

*Feature Selection Pipeline*:

S0. *Input Data*: 49 melanoma cell lines with 19K gene expression features.
S1. Apply the Fisher Scoring method (univariate correlation-based method extended to multiple features) on *Input Data* and select the top 500 gene expressions with the highest scores.
S2. Perform the SVM-RFE+CBR method (Yan and Zhang, 2015) on *Input Data* and select the top 500 gene expressions based on the SVM-RFE+CBR ranking.
S3. *Reduced Data*: Combine the top 1,000 gene expressions obtained in (S1) and (S2). After eliminating duplicates, the resulting reduced data contains 49 cell lines with 928 gene expressions.
S4. Perform 500 randomized trials of SFFS wrapped around KNN on *Reduced Data* and record the genes that occur most often in the resulting optimal 20 feature subsets.
S5. *Output Data*: 49 cell lines with the top 15 genes obtained from the randomized sequential trials of k-Nearest Neighbors in (S4).

The Fisher Score has been used in several studies on genetic datasets due to its computational simplicity (Yang et al., 2016; Sun et al., 2018; Li and Xu, 2019). The Fisher Score of the *i*th feature is calculated as


$$S_{i}=\frac{\sum_{j}n_{j}{\left(\mu_{ij}-\mu_{i}\right)}^{2}}{\sum_{j}n_{j}\sigma_{ij}^{2}},$$

where μ_*ij*_ and σ_*ij*_ are the mean and standard deviation of the *i*th feature in the *j*th class, respectively. *n*_*j*_ is the number of instances in the *j*th class while μ_*i*_ is the mean of the *i*th feature (Aggarwal, 2014). This produces a rank among the features. However, being a function of single features, it does not capture any relationships between features. Support Vector Machine Recursive Feature Elimination (SVM-RFE), originally proposed by Guyon et al. (2002), is an embedded feature selection algorithm that uses criteria derived from the coefficients in SVM models to assess feature importance. The algorithm features a backward search that begins with the full feature set and recursively removes the features with the lowest importance. SVM-RFE+CBR is an extension to SVM-RFE that addresses the problem that the importance of highly correlated features is likely to be underestimated. SVM-RFE+CBR introduces a correlation bias reduction step to reintroduce a representative feature from a correlated group that has been possibly removed entirely due to correlation bias. This is important to capture as our full feature set has a large number of correlations. SFFS, first introduced in Pudil et al. (1994), is a forward search algorithm that begins with the empty set and incrementally adds features based on the objective function, in this case, the classification performance of k-nearest neighbors on the test set. The candidate feature set is *floating* in the sense that after each forward step, the algorithm performs conditional backward steps. It removes features starting with the one that increases the objective function the most if any such feature exists. The backward steps will continue as long as the objective function increases. The algorithm terminates when the floating subset of features reaches the desired size. This algorithm adds and removes features one at a time, so it is not practical to apply it to all 19 K features.

**Figure 4:**
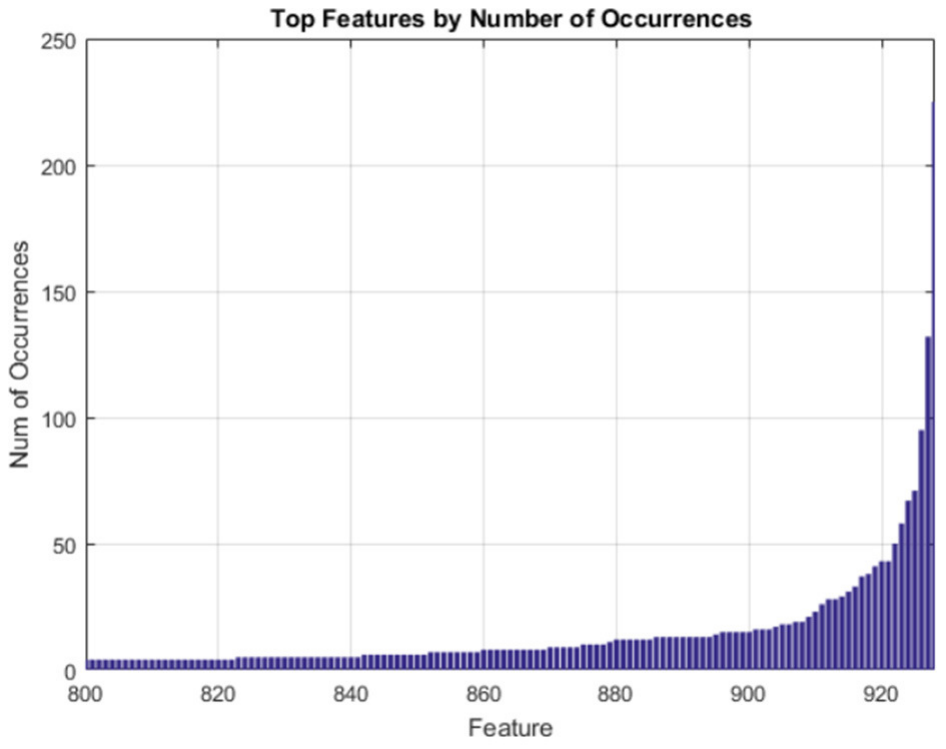
Gene expression features ranked by frequency of occurrence over 500 randomized trials of SFFS.

The features which occurred most often in our randomized trials of SFFS are the features we regard as the most compact and generalizable descriptors of our clusters. Multiple randomized trials help us avoid results emergent from artifacts present in any single run of the feature selection pipeline. The drop in frequency of occurrence among the most frequent genes is very steep as shown in Figure 4.

### 3.2. Top 15 Most Significant Genes

**Figure 5:**
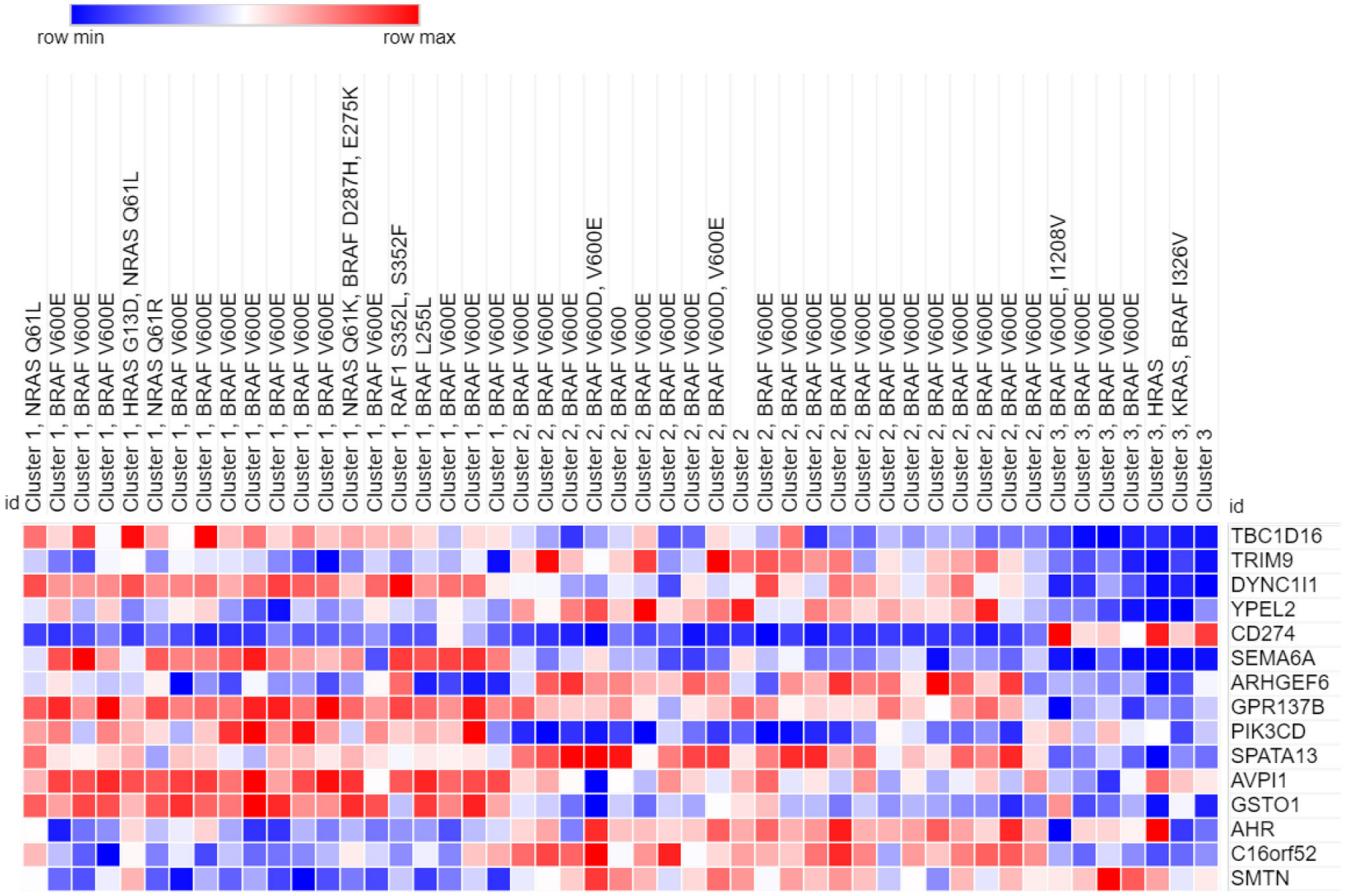
Heatmap of 49 melanoma cell lines with top 15 genes.

Our analyses showed that only the top 15 genes obtained from the SFFS algorithm in (S4) were sufficient for perfect or near-perfect classification performance across various classifiers. These genes are displayed in order of frequency (importance) in Table 1. The heatmap of 49 cell-lines with the expression levels of the top 15 genes is shown in Figure 5.

**Table 1:** Top 15 genes for CCLE skin cell line tumors.

TBC1D16	SEMA6A	AVPI1
TRIM9	ARHGEF6	GSTO1
DYNC1I1	GPR137B	AHR
YPEL2	PIK3CD	C16ORF52
CD274	SPATA13	SMTN

Of these 15 genes, some have links to melanoma prognosis. While other genes have links to other cancers, their relationship to melanoma was never previously shown. Here are brief descriptions of some of the genes which have recently been linked to various cancers and have the potential to serve as prognostic biomarkers:

- **TBC1D16** a GTPase-activating protein for RAB family proteins is suggested to regulate EGFR in melanoma as a result of a hypomethylation event. This may confer poor survival but at the same time may increase BRAF and MEK response (Vizoso and et al., 2015). Recently it has been linked to epithelial ovarian cancer (EOC) as a positive predictive marker for favorable outcomes for EOC. Impact is thought to be conferred by effects on angiogenesis through vascular endothelial growth factor (VEGF) signaling (Yang et al., 2018). Rodger et al. (2019) have also reported epigenetic changes in DNA methylation in TBC1D16. Loss of methylation was noted in metastatic melanoma when compared to primary tumors. Similar changes in methylation between primary and metastatic tumors were noted in other cancers such as breast, prostate, and colorectal cancer, suggesting that TBC1D16 may be an epigenetic driver for tumor metastasis (Rodger et al., 2019).
- **TRIM9** Tripartate motif containing 9 is part of the TRIM family of proteins, several of which are associated with oncogenesis. TRIM9 is expressed normally in brain neurons and recently has been linked to lung cancers. TRIM9 is an ubiquitin ligase and may regulate tumor proliferation through VEGFA and angiogenesis (Wang et al., 2016).
- **DYNC1I1** Cytoplasmic Dynein 1 protein regulates intracellular transport and mitotic spindle localization. These activities may promote cell migration and cell cycle progression. Recently it has been reported to play a role in gastric cancer progression (Gong et al., 2019).
- **CD274** Gene codes for Programmed death-ligand 1 (PD-L1) which is currently the target of several large trials showing substantial benefit with anti-PD-L1 for late-stage melanoma (Ascierto and Marincola, 2015). PD-L1 expression is associated with overall better response rate and survival when treated with the PD-1 checkpoint inhibitors. PD-L1 is the ligand for PD-1 receptors on T-cells and suppresses anti-tumor T-cell mediated immune responses. CD274/PD-L1 expression is reported in TP53-mutated melanoma, non-small cell lung cancer, colorectal cancer, and renal cell carcinoma (Huang et al., 2018; Thiem et al., 2019).
- **PIK3CD** The catalytic subunit of phosphoinositide 3-kinase (PI3K) is encoded by PIK3CD. PIK3CD has been reported to be overexpressed in colorectal cancer (CRC) and associated with poor survival and may be a prognostic biomarker for CRC but no relation to melanoma has been observed to date (Chen et al., 2019).
- **GSTO1** Glutathione S-transferase omega-1 polymorphisms are associated with the increased risk of developing breast and liver cancer and have very recently been implicated in squamous cell, colorectal, and melanoma as a modulator of cell growth and immune response (Xu et al., 2020). Elevated expression levels of GSTO1 have been correlated with drug resistance.
- **AHR** The aryl hydrocarbon receptor initially was studied for its role in response to environmental pollution and toxicity. In recent studies it has been identified as playing a key role in malignant cell progression, tumor aggression, and poor prognosis (Wang et al., 2021).

### 3.3. Validation of the Top 15 Genes

**Figure 6:**
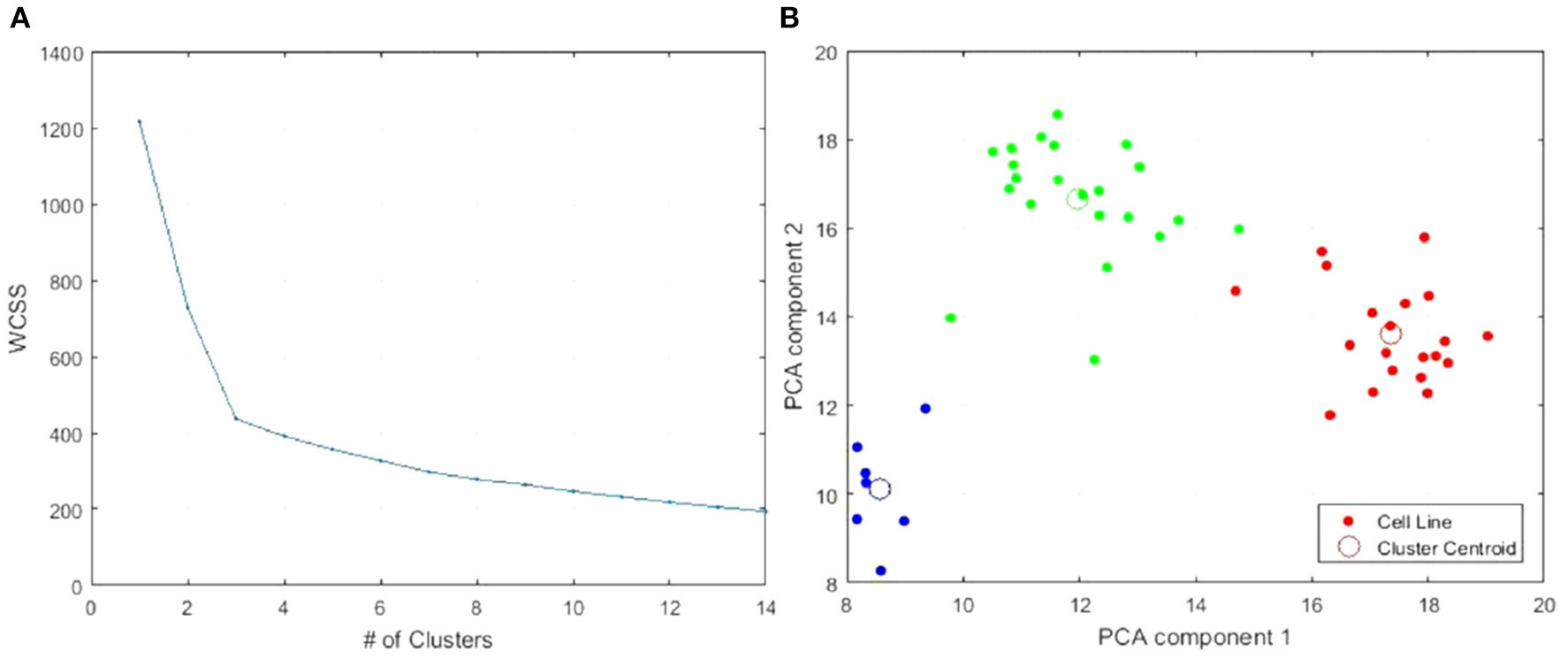
Clustering of the 49 CCLE skin cancer tumors using the top 15 genes only. **(A)** WCSS error. **(B)** PCA plot of 3-clusters using only the top 15 genes: Cluster 1, Cluster 2, Cluster 3. The colors are used to distinguish each Cluster. This color scheme is carried through on Table 2 and 5 to make it easy to distinguish each cluster.

We reran *k*-means++ clustering on the 49 CCLE skin cancer tumors with the top 15 genes only. We found the elbow-point at exactly three clusters was greatly accentuated as shown in Figure 6A compared to Figure 3A. Figure 6B presents the PCA plot of the three clusters using the top 15 genes. All cell lines clustered together in the same way as when considering all original 19 K genes as previously shown in Figure 3B.

Heatmap of the clustered cell lines with top 15 genes is presented in Figure 5. Cluster membership and genomic characterization of the reduced data is shown in Table 2.

**Table 2:** Cluster membership and genomic characterization of cell lines.

**Cluster 1 cell lines**	**Mutations**	**Cluster 2 cell lines**	**Mutations**	**Cluster 3 cell lines**	**Mutations**
IPC298	NRAS Q61L	COLO829	BRAF V600E	LOXIMVI	BRAF V600E I1208V
K029AX	BRAF V600E	SKMEL24	BRAF V600E	RPMI7951	BRAF V600E
SKMEL3	BRAF V600E	HS695T	BRAF V600E	WM793	BRAF V600E
MALME3M	BRAF V600E	WM115	BRAF V600D V600E	IGR39	BRAF V600E
MELJUSO	HRAS G13D NRAS Q61L	COLO800	BRAF V600	CJM	HRAS
SKMEL2	NRAS Q61R	UACC62	BRAF V600E	GRM	KRAS BRAF I326V
COLO679	BRAF V600E	A375	BRAF V600E	BJHTERT	N/A
SKMEL5	BRAF V600E	HS294T	BRAF V600E		
UACC257	BRAF V600E	WM2664	BRAF V600D, V600E		
IGR37	BRAF V600E	COLO849	N/A		
MELHO	BRAF V600E	C32	BRAF V600E		
G361	BRAF V600E	RVH421	BRAF V600E		
COLO741	BRAF V600E	HT144	BRAF V600E		
SKMEL30	NRAS Q61K BRAF D287H E275K	SKMEL31	BRAF V600E		
SKMEL1	BRAF V600E	A101D	BRAF V600E		
COLO792	RAF1 S352L S352F	COLO783	BRAF V600E		
MEWO	BRAF L255L	WM983B	BRAF V600E		
IGR1	BRAF V600E	MDAMB435S	BRAF V600E		
SKMEL28	BRAF V600E	WM1799	BRAF V600E		
SH4	BRAF V600E	WM88	BRAF V600E		
		COLO818	BRAF V600E		
		A2058	BRAF V600E		

In all 20 of the cells lines found in Cluster 1, there is an identified mutation in a gene that is characteristic of the canonical MAPK pathway. This includes genes such as NRAS, HRAS, BRAF, and RAF1. In fact, 14 out of 20 cell lines express the classic BRAF V600E mutation, which is a missense mutation found in many neoplasms. Furthermore, patients with BRAF V600E mutated melanomas respond to FDA-approved BRAF inhibitors, such as atezolizumab. A dependency on the essential SOX10 transcription factor in CRISPR and/or RNAi loss-of-function screens is shown in 13 out of 20 Cluster 1 cell lines.

In our identification of the top 15 genes that helped identify the three clusters, TBC1D16, DYNBC1l1, SEMA6A, GPR137B, PIK3CD, AVPl1, and GST01 are found to be high expressing genes (relative to the other clusters) for Cluster 1. TRIM9, ARHGEF6, C16orf52, and SMTN are also lower expressing genes for Cluster 1.

There are 22 cell lines that partitioned into Cluster 2. Of these, 21 are catalogued for mutations. All 21 are positive for the BRAF V600E mutation. We observe that none of these cell lines have mutations in any of the RAS genes, which stands out from the cells in Cluster 1.

Examples, of preferentially essential genes in some of the cell lines in Cluster 2, as determined by CRISPR and/or RNAi screens, included BRAF, MAPK1, SOX10, MDM2, and DUSP4.

Based on our analysis, TRIM9, YPEL2, ARHGEF6, SPATA13, AHR, C16orf52, and SMTN are high expressing genes present in the cell lines of Cluster 2. Of this group, RIM9, ARHGEF6, C16orf52, and SMTN are low expressing genes in cell lines of Cluster 1.

There are seven cells lines that partitioned into Cluster 3. One is a fibroblast cell line and is not represented in the Cancer Dependency Map portal from the Broad Institute (depmap.org/portal). Of the six represented cells lines, five had mutations in the BRAF gene: four with the typical BRAF V600E mutation and one with BRAF I326V, which has been found in other cancers (colorectal, breast, and lymphoid).

Only one of the six cell lines show preferentially dependent genes in the MAPK pathway. None of the other five out of six cell lines showed dependency on proteins from the MAPK pathway in Cluster 3.

Only CD274 is found to be high expressing in the cell lines in Cluster 3. However, Cluster 3 has the most representatives of the 15 genes as low expressing genes, including TBC1D16, DYNC1l1, YPEL2, SEMA6A, GPR137B, SPATA13, and AVPl1.

To validate the discriminating power of the top 15 genes among our three clusters we applied various classification techniques on 49 cell lines with the 15 genes. Table 3 shows the perfect or near-perfect accuracy of different classification algorithms implemented in the machine learning software WEKA (Hall and et al., 2009).

**Table 3:** Classification accuracy for top 15 genes using six different classification techniques.

**Cluster**	**Multilayer perceptron (%)**	**Logic regression (%)**	**Naive Bayes multinomial (%)**
**1**	100	100	100
**2**	100	100	100
**3**	100	85.70	100
Average	100%	95.23%	100%
**Cluster**	**k-Nearest** **Neighbor**	**Logic model** **tree**	**Random** **forest**
**1**	100	85	100
**2**	100	100	100
**3**	100	100	100
Average	100%	95%	100%

### 3.4. Decision Tree Classification Model

We built a Decision Tree classification model on data consisting of the 49 cell lines with the top 15 genes to obtain combinatorial patterns of gene expression that led to the separation of the clusters. Decision Tree is a non-parametric supervised learning method used for classification and regression. The goal is to create a model that predicts the value of a target variable by learning simple decision rules inferred from the data features (Quinlan, 1993). The decision tree model shown in Table 4 consists of just one pattern for Cluster 1, one for Cluster 2, and two patterns for Cluster 3. The decision rules include only three genes: GSTO1, TBC1D16, and TRIM9.

**Table 4:** Tabular description of the decision tree classification model.

**Cluster**	**Decision tree rules**
**1**	*GSTO*1≥12.7765 & *TBC*1*D*16≥7.28245
**2**	*GSTO*1 <12.7765 & *TRIM*9≥5.03635
**3**	*GSTO*1≥12.7765 & *TBC*1*D*16 <7.28245
**3**	*GSTO*1 <12.7765 & *TRIM*9 <5.03635

*Each rule specifies required levels of gene expression (as measured in the CCLE database) to fall into the given cluster. There was only one pattern that defined Clusters 1 and 2, respectively. Cluster 3 was defined by two patterns, as shown*.

We evaluated the performance of the Decision Tree model through 10 times *k*-folding (10-folding in this case) cross-validation experiments: Randomly partition the data into *k* = 10 approximately equal parts. Designate one of these subsets as a test set. Build a model on the remaining *k*−1 = 9 subsets that form the training dataset. Then, evaluate classification performance on the test set. Finally, average performance while using each fold as the test set once. Table 5 displays the cross-validation accuracy of the Decision Tree.

**Table 5:** Cross-validation accuracy of the decision tree classification model.

**Average accuracy**	**Cluster 1 (%)**	**Cluster 2 (%)**	**Cluster 3 (%)**
98.33	95	100	100

## 4. Drug Response Analysis

Analysis of the drug response data provided by the CCLE suggests that these 15 genes also play a role in predicting drug responses. The CCLE reports drug response in a subset of the cancer cell lines as ActArea—the area above the fitted dose-response curve—and includes responses across 24 different anti-cancer drugs. Of these 24 anti-cancer drugs, we focused on two MEK inhibitors. These are believed to be crucial in the treatment of melanoma. Upon analyzing the mean response of these two drugs across all three of our clusters separately, we found a decreasing response from cluster 1 to cluster 3 across both drugs as shown in Table 6. Given that our study illuminates what genes distinguish our clusters, one can reasonably hypothesize that these same genes are possibly responsible for this decreased response. However, further analysis is needed before making any conclusions in regards to this.

**Table 6:** Drug response analysis for the 49 melanoma cell lines.

**Drug analysis**		**AZD6244 (MEK) ActArea**	**PD-0325901 (MEK) ActArea**
Cluster 1	Sample size	13	13
	Mean	2.9335	4.1911
	Standard deviation	1.0908	1.3765
Cluster 2	Sample size	16	16
	Mean	2.6125	3.7534
	Standard deviation	1.1446	1.5811
Cluster 3	Sample size	4	4
	Mean	1.5511	2.1695
	Standard deviation	0.8569	0.8631

## 5. Conclusion

Through a machine learning pipeline of clustering and feature selection, our study identifies 15 genes whose levels of expression categorize melanoma into three genomic clusters. A Decision Tree model needs only three genes—GSTO1, TBC1D16, and TRIM9—to reliably classify nearly all of the cell lines into their respective clusters. These results shed some light on the globally relevant genetic intravariability among melanoma cell lines. These results may indicate extra considerations in choosing a cancer therapy in addition to the treatment of the BRAF mutation that exists in 42/48 (one of the 49 cell lines used in this study lacks analysis) of the CCLE cell lines across all clusters (Ghandi et al., 2019). Further research may include applying our same feature selection pipeline with drug response as an outcome variable to discover key gene expression patterns for predicting drug response levels.

## Data Availability Statement

Publicly available datasets were analyzed in this study. This data can be found at: https://portals.broadinstitute.org/ccle/.

## Author Contributions

MS, LM, DC, and ES are senior co-authors who designed and supervised the entire project. BK, RB, and TB participated in the study design and performed all data analysis. BK, MS, and LM developed the manuscript. NC and AP were involved gene literature review. All authors contributed to the article and approved the submitted version.

## Funding

BK, RB, NC, and AP were supported by National Science Foundation (NSF) Research Experience for Undergraduates (REU) Grant no. 1359341. Publication of this article was funded in part by the Open Access Subvention Fund and the John H. Evans Library.

## Conflict of Interest

The authors declare that the research was conducted in the absence of any commercial or financial relationships that could be construed as a potential conflict of interest.

## Publisher's Note

All claims expressed in this article are solely those of the authors and do not necessarily represent those of their affiliated organizations, or those of the publisher, the editors and the reviewers. Any product that may be evaluated in this article, or claim that may be made by its manufacturer, is not guaranteed or endorsed by the publisher.
